# Thermal and Oxidative Aging Effects of Polyamide-11 Powder Used in Multi-Jet Fusion

**DOI:** 10.3390/polym15102395

**Published:** 2023-05-21

**Authors:** Chrysoula Pandelidi, Ryan Blakis, Kok Peng Marcian Lee, Stuart Bateman, Milan Brandt, Mladenko Kajtaz

**Affiliations:** Centre for Additive Manufacturing, School of Engineering, RMIT University, GPO Box 2476, Melbourne, VIC 3001, Australiastuart.bateman@rmit.edu.au (S.B.)

**Keywords:** polyamide-11, thermal aging, mechanical performance, powder aging, powder bed fusion, additive manufacturing

## Abstract

The transition of additive manufacturing (AM) from a technique for rapid prototyping to one for manufacturing of near net or net components has been led by the development of methods that can repeatedly fabricate quality parts. High-speed laser sintering and the recently developed multi-jet fusion (MJF) processes have seen quick adoption from industry due to their ability to produce high-quality components relatively quickly. However, the recommended refresh ratios of new powder led to notable amounts of used powder being discarded. In this research, polyamide-11 powder, typically used in AM, was thermally aged to investigate its properties at extreme levels of reuse. The powder was exposed to 180 °C in air for up to 168 h and its chemical, morphological, thermal, rheological, and mechanical properties were examined. To decouple the thermo-oxidative aging phenomena from AM process related effects, such as porosity, rheological and mechanical properties characterisation was performed on compression-moulded specimens. It was found that exposure notably affected the properties of both the powder and the derived compression-moulded specimens within the first 24 h of exposure; however, consecutive exposure did not have a significant effect.

## 1. Introduction

Polyamides (PA) are increasingly being introduced as the material of choice for end-use products due to their good mechanical performance and high barrier properties to liquids and gasses [1,2]. As such, it is important that their aging behaviour is understood in pre- and post-fabrication stages, as degradation can negatively affect mechanical performance [3,4,5,6,7]. Here, we define pre-fabrication aging as the degradation of the material prior to its processing for final component manufacturing and post-fabrication the aging of the final component or the aging of the test specimens after their manufacturing. Industries that use traditional manufacturing methods, such as injection moulding, would only be concerned with post-fabrication aging behaviour as a result of heat treatment [3], as any aging caused by the fabrication process or the operating environment can be well understood, whereas pre-fabrication aging effects are becoming continuously more relevant with the expansion of powder bed fusion (PBF) additive manufacturing (AM) technologies, where the previous aging of the starting materials is not always consistent due to powder reuse. During PBF AM, a thin layer of powder is deposited on a substrate and then selectively melted following a 2D slice of a computer aided design (CAD) model. This process is repeated until the CAD 3D model has been constructed [8]. The most common PBF processes for polymers are selective laser sintering (SLS) and multi-jet fusion (MJF), the most recently developed process by HP [9]. Powders used in PBF AM are exposed to high ambient temperatures to increase particle fusion efficiency. This allows for shorter exposure times to the energy source, infrared lamps or laser, and consequently increasing productivity. However, only a portion of the exposed powder is fully melted during processing [10]. The remaining powder would ideally be reused in the following fabrication cycle. As these powders are exposed to high temperatures, and in some cases in air rather than an inert atmosphere, thermal–oxidative-based degradation is presumed to take place enforcing fixed refresh rates and in turn restricting their full recycling potential [7].

Studies on the degradation behaviour of polymeric components cannot be directly transferred to behaviour of powders because powders have significantly higher specific surface [7] and therefore need to be studied separately. At the same time, the extent and manifestation of degradation is dependent on polymer structure, impurities, polymerisation method, additives, and end groups among other things [3]. This means that observations are not linearly transferable among different grades of the same material or even the same material as sourced from different manufacturers. The thermal aging of PA powders is generally manifested through cracks in the powder particles, an increase in melt viscosity [11], widening of the melt peak, and increase in melt temperature [12]. These are the result of cross-linking, which in PAs is predominant in the absence of oxygen. In the presence of oxygen, thermal–oxidative degradation takes place, where through a series of chemical reactions chain scission predominantly takes place. However, these chain reactions may be stopped by the recombination of two alkyl radicals, in which case crosslinking occurs. The final aging observations are dependent on the grade of PA and the conditions of aging [7].

There is a variety of PA grades available in the market, each with a distinct range of characteristics and applications. Of these, PA11 and PA12 are the two more broadly used grades for PBF AM processing. The more established PA12 has been investigated in its mechanical [13,14,15,16] and thermal [11,12,15,16,17,18,19] behaviour. Pre-fabrication aging of PA12 powder has also been investigated for effects in PBF AM processes [7,15], while less data is available on PA11. PA11 has extensively been used in hydrocarbon transport pipes [20] and flexible risers [4] in oil and gas industry applications, air brake systems and fuel lines in the automotive industry [2], due to its good fluid resistance, while its recent introduction as an AM material has seen it expand in the prosthetics and orthotics industry [9]. As a result, of particular interest has been the observation of hydrolysis, to which hydrophylic thermoplastic polymers are susceptible [4,20]. Post-fabrication thermal–oxidative aging of PA11 films has previously been investigated by Okamba-Diogo et al. in a series of publications where they found a correlation between carbonyl formation and discolouration, established that chain scission predominates over crosslinking as the aging mechanism, and that thermal–oxidative aging can lead to embrittlement [2,5,21]. 

The mechanical performance of MJF-manufactured PA11 specimens was previously investigated by Lee et al. [22], while Pandelidi et al. [9] previously highlighted the need for pre-fabrication aging characterisation of PA11 in order to understand the effect of powder reuse in MJF. Therefore, this research investigates the effects of thermal–oxidative aging on PA11 powder used as the starting material for specimen fabrication in MJF. Thermal and physicochemical analysis of the aged powder is performed, and to avoid any AM process related effects such as porosity, specimens are fabricated through compression moulding for rheological and mechanical characterisations.

## 2. Materials and Experimental Methods

### 2.1. Material and Aging Procedure

PA11 powder as used in MJF was supplied by the original manufacturer with the commercial name “HP 3D High Reusability PA11”. The as-received powder was then placed in sealed metallic canisters and in an air-forced oven at 180 °C for 0, 24, 48, 72, 96, and 168 h. The powder and resulting specimens are henceforth denoted as PA11_0C, PA11_2C, PA11_4C, PA11_6C, PA11_8C, and PA11_14C, respectively. The powder was not dried separately before the aging process. 

The aging conditions are generally selected based on specific service conditions and as a result are not easily transferable. The environmental conditions (presence of oxygen or water) as well as the selected temperature and duration of exposure are expected to affect the observed aging behaviour [23]. In the case of PA11, the presence of stabilisers has also been found to result in variations in its kinetic behaviour [5]. In this research, the aging conditions were chosen so that to represent the temperature which the unfused powder is exposed to during MJF, and the time intervals were chosen to represent approximately two processing cycles (each cycle is approximately 12 h).

The effects of this thermal–oxidative aging process on the thermal and physicochemical properties of the powder as well as the rheological and mechanical properties of the resulting specimens were then investigated.

### 2.2. Fourier-Transform Infrared Spectroscopy

Fourier-transform infrared spectroscopy (FTIR) was performed in the infrared region of 4000–600 cm^−1^ using a PerkinElmer Spectrum 100 FTIR Spectrometer in ATR mode. The spectra for the powder specimens were analysed for all aging stages of the powder. Each spectrum was based on 32 scans at 4 cm^−1^ resolution and 0.2 cm/s scan speed.

FTIR was performed to identify oxidative phenomena that may have taken place during aging. The carbonyl index was calculated as A1718 cm^−1^/A1193 cm^−1^ as the spectra around 1193 cm^−1^ were found to remain unaffected by thermo-oxidative aging phenomena [24].

### 2.3. X-ray Photoelectron Spectroscopy

A K-alpha X-ray photoelectron spectrometer (XPS) from ThermoFisher Scientific was used to detect the elements present on the surface of the aged PA11 powder particles. The data was further processed using CasaXPS software. A Gaussian/Lorentzian peak shape was selected for the curve fitting of the carbon and oxygen peaks to quantify the changes in nitrogen and oxygen contents.

### 2.4. Powder Size Distribution

Powder size distribution (PSD) measurements were conducted using a Malvern Mastersizer 3000 fitted with an Aero S dry powder dispenser. The results presented are the average of five measurements for each of the powder stages.

### 2.5. Scanning Electron Microscopy

The morphology of the aged powder particles was assessed through scanning electron microscopy (SEM). Powder particles were mounted onto the holder using carbon tape. They were then coated with 3 nm Iridium for imaging. SEM was performed using a Quanta 200 by FEI using 30 kV accelerating voltage and spot size of 5 in high vacuum.

### 2.6. Thermogravimetric Analysis

The degradation temperature (T_d_) of the different stages of PA11 was measured through thermogravimetric analysis (TGA) using a high-temperature NETZSCH STA 449 Jupiter. The specimens were isothermally held at 25 °C for 3 min and then the temperature was increased at a rate of 10 °C/min up to 1000 °C in a N_2_ flow rate of 20 ml/min. The T_d_ was noted at 5% weight loss and onset degradation for all specimens.

### 2.7. Differential Scanning Calorimetry

Differential scanning calorimetry (DSC) was used to observe potential variances in melting (T_m_) and crystallisation (T_c_) temperatures for all stages of PA11. A DSC 250 by TA Instruments was used for this purpose. The specimens were initially stabilised at 25 °C for 3 min, then the temperature was increased to 360 °C at a rate of 5 °C/min, held at 360 °C for another 3 min and then cooled down to 25 °C at a rate of 5 °C/min. This cycle was repeated twice for each specimen. Two specimens were tested for all stages of the powder.

DSC was also used to calculate the changes in crystallinity for all stages of PA11 powder based on

$$X_{c}=\frac{{\Delta H}_{m}}{{\Delta H}_{0m}}\cdot 100\%$$

where ΔH_m_ is the heat of each sample normalised with respect to its mass and ΔH_0m_ = 189.05 J/g as a reference for 100% crystalline PA11 [25].

### 2.8. Specimen Fabrication

**Figure 1:**
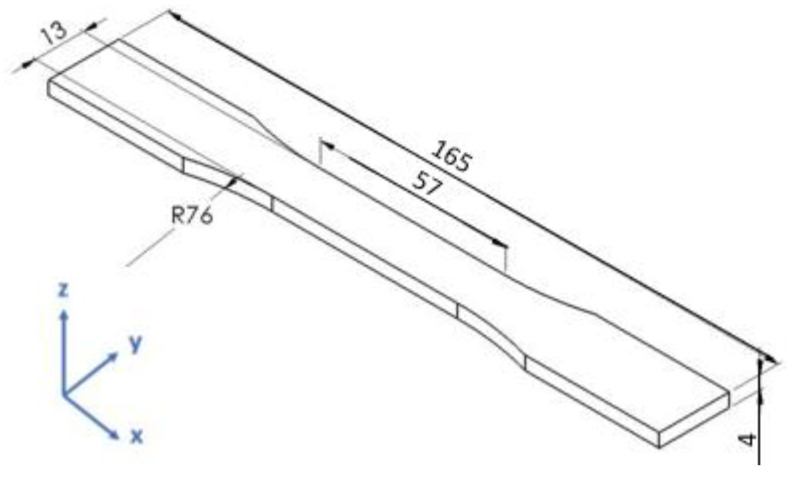
Drawing of tensile specimen (Type I) according to ASTM D638 standard. Dimensions in mm.

Powder was collected from the metallic canisters at each time interval and used for the fabrication of Type I specimens according to ASTM D638-14 standard [26], as shown in Figure 1, rectangular specimens of 4 × 2 × 35 mm^3^, and disks with 25 mm diameter and 2 mm thickness. The specimens were compression-moulded using a hot press at 250 °C under 100 kN of pressure for 5 min. The hot press used water cooling to cool to approximately 40 °C over 10 min. The mould was then removed from the hot press after the cooling cycle, and subsequently the specimens were removed from the mould. For the specimens that could not be fabricated immediately after the powder aging cycle, the powder was further dried prior to compression moulding at 80 °C for at least 5 h in a vacuum oven, to avoid further oxidative effects.

Type I specimens were visually inspected for defects in the gauge section and only those that did not contain any visible defects, such as cavities, were tested. Five Type I specimens were fabricated for each aging stage of the powder.

### 2.9. Parallel Plate Rheology

The fabricated disks were used to perform parallel plate rheology using a Discovery HR3 rheometer by TA Instriments. Experiments were performed in oscillation with frequency sweep between 0.1 and 100 rad/s at 250 °C and 3% strain. Storage (G′) and Loss (G″) modulus as well as complex viscosity (η*) measurements were obtained within this region for all specimens.

### 2.10. Dynamic Mechanical Analysis

Dynamic mechanical analysis (DMA) was performed on rectangular compression-moulded specimens using a DMA Q800 by TA Instruments. One specimen per aging stage was conditioned for at least 24 h before testing at 21 °C and 50% humidity. Specimens were tested in single cantilever mode with a fixed frequency of 1 Hz, amplitude of 20 μm, and an increasing temperature from 35 to 150 °C at a rate of 2 °C/min.

### 2.11. Tensile Testing

The fabricated tensile specimens were tested under tension using an Instron 5900R equipped with a 5 kN load-cell. The tests were completed following ASTM D638-14 standards [26] at 5 mm/min crosshead speed and ambient temperature regulated at approximately 21 °C. The elongation at break (ε_Break_) was measured based on the crosshead displacement from the initial 115 mm set distance between the grips. Young’s Modulus (E_Tensile_) was calculated from the slope of the stress–strain curves between 1 and 2% strain. Five specimens were tested for each aging stage to ensure repeatability. All specimens were conditioned in a chamber regulated at 21 °C and 50% humidity for more than 24 h prior to testing.

## 3. Results and Discussion

### 3.1. Physicochemical Observations

Thermal–oxidative degradation due to exposure of polymers to oxygen and high temperatures is usually observed in changes in both physical and chemical characteristics [3,27]. Physical degradation is caused by changes in the order of molecules and is manifested through entanglement, post-crystallisation, and relaxation and is a reversible process. On the other hand, chemical degradation is the result of changes in the chemical structure of the polymer such as branching, crosslinking, and chain scission [7]. Both physical and chemical aging often happen in parallel and it is not always clear which one is dominating the properties of the polymer [28].

**Figure 2:**
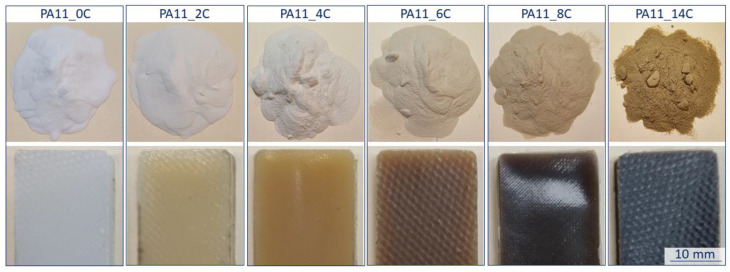
Colour of powder (**top**) and resulting specimens (**bottom**) for all aging stages.

Colour change has been identified as one of the easiest ways to observe chemical aging and is understood to be common in PA when exposed to air [5,29]. This phenomenon has been previously explained to be a result of oxidation reactions [30]. Figure 2 shows the colour of the powder and corresponding fabricated specimens at each aging stage. Both show noticeable discolouration from the white colour of the as-received PA11_0C to a brown for PA11_14C. This colour change, even though noticeable in the powder, is more pronounced in the compression-moulded specimens. Unlike observations in post-fabrication aged specimens, where oxidation depth has been found to be limited to the surface and temperature dependent [2], the discolouration of the specimens in this research was uniform throughout their thickness. This was expected as the starting material was aged instead of the fabricated specimen confirming that the observed discolouration is a result of oxidative reactions, which, in this research, would have taken place on the surface of the powder particles. It is generally accepted that oxidative reactions are more pronounced on the exposed surface of the material [23]. Pliquet et al. [31] explained that these diffusion-limited oxidation profiles occur because the oxidation rate increases at a greater rate than oxygen diffusion. This, in turn, means that oxygen is consumed at the exposed surface before it penetrates to the core [31]. 

**Figure 3:**
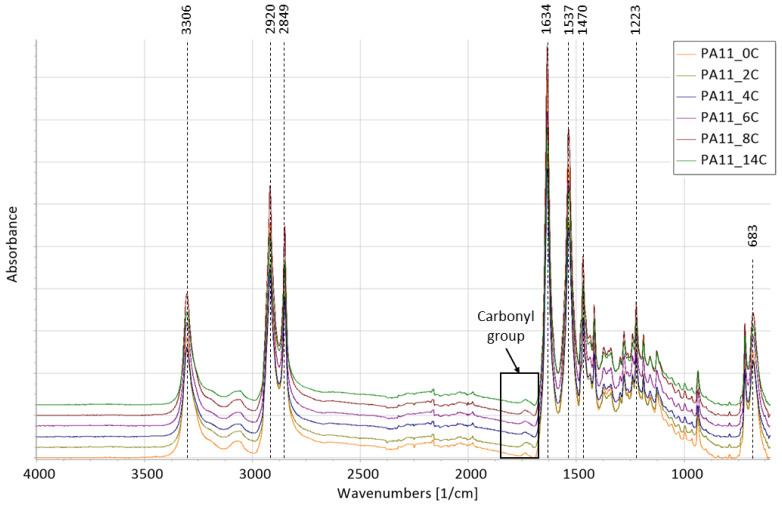
FTIR spectra for all stages of PA11 powder.

Chemical degradation as a result of oxidation has previously been investigated through FTIR [32]. The spectra of the as received and aged PA11 powder at all aging stages are presented in Figure 3. Similar to observations by Tey et al. [33]:

- The absorbance at 3306 cm^−1^ reflects N-H stretching,
- Asymmetric and symmetric stretching of CH_2_ is found at 2920 and 2849 cm^−1^, respectively;
- The absorbance at 1634 cm^−1^ indicates C=O stretching (Amide I);
- The absorbance at 1537 cm^−1^ may be assigned to C-N stretching and C=O in-plane bending;
- The absorbance at 1470 cm^−1^ can be assigned to C=O and N-vicinal CH_2_ bending
- C-N stretching is found at 1223 cm^−1^;
- The absorbance at 683 cm^−1^ may be assigned to CONH out-of-plane deformation (Amide V).

Degradation is often manifested through increase in absorbance of IR spectra in the carbonyl area around 1750–1700 cm^−1^ [2,20,23,24,30]. Eriksson et al. [23] explained that this observed growth on the absorption bands in the carbonyl group might be the result of differences in the degree of hydrogen bonding, the formation of additional carbonyl groups, differences in the conformation of those carbonyl groups, as well as their temperature dependence. Other products of the PA oxidation cycle may include carboxylic acids at around 1715 cm^−1^ and aldehydes at around 1705 cm^−1^ [18]. Oxidation is discussed to be related to discolouration, such as that observed in Figure 2, due to the presence of chromophoric groups [21,30]. Li and Hu [29], through a comprehensive analysis of PA6, proposed that the chromophore structure is α-keto carboxyl at 1730 cm^−1^. Sang et al. [30] expressed that chromophoric groups such as ketones at approximately 1710 cm^−1^ may explain the colour transitions. Table 1 shows our calculated values for the carbonyl index where an overall increasing trend with exposure time may be noted. Similar trends have previously been observed by Pliquet et al. [31,34], who quantified the absorbance at both 1734 cm^−1^, where imides are allocated [31], and 1712 cm^−1^ for ‘bonded’ carboxylic acids of PA6,6 [34]. The increase in the carbonyl index is more pronounced between PA11_0C and PA11_2C, where slight variances were observed until PA11_6C, and then a notable increase between PA11_6C and PA11_8C.

**Figure 4:**
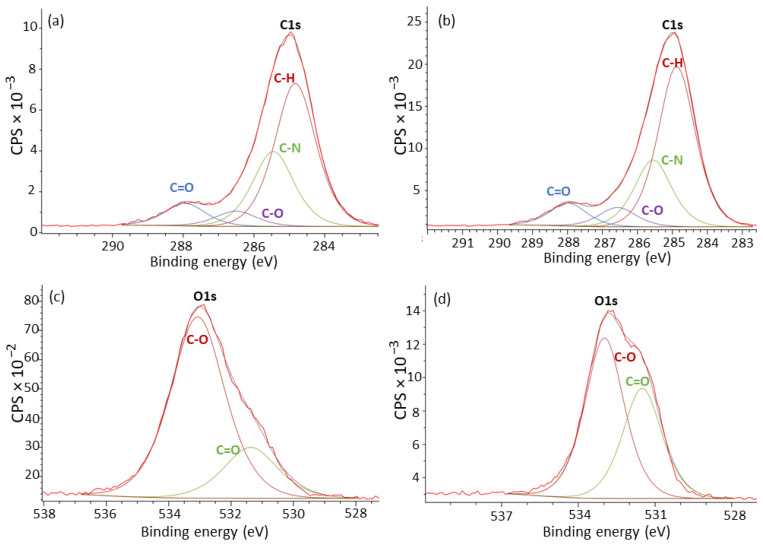
XPS analysis of high-resolution spectra of carbon chemical state for (**a**) PA11_0C and (**b**) PA11_14C and oxygen chemical state of (**c**) PA11_0C and (**d**) PA11_14C.

To investigate this further and in more detail, we conducted an analysis of the chemical states of the surface of all powders using XPS. Peaks at 532, 340, and 285 eV corresponding to the oxygen, nitrogen, and carbon, respectively, were found as well as minor peaks at 156 and 104 eV manifesting the presence of silicon bonds. Silicon is assumed to be present due to the containment of inorganic powder additives which aim to improve oxidation resistance and powder flowability [33]; however, should be noted that contamination by silicon could have occurred by the powder storage containers or the laboratory environment as well. Figure 4 shows the fitted curves for carbon and oxygen for PA11_0C and PA11_14C where the decrease in C-N content in the carbon peaks and increase in C=O content in the oxygen peaks of PA11_14C are evident. This establishes that oxidation has indeed taken place on the surface of the exposed powders.

Table 1 presents the contents of the expected chemical bonds in all powders as well as the calculated ratio of nitrogen contained in the amide groups in PA11 to the ketones (C=O). It appears that the content of ketones is increased over that of the amide groups with exposure time. These changes can be attributed to thermal–oxidative degradation [23] and may explain the change in colour shown in Figure 2. It has previously been explained that an oxidative attack favourably takes place in the α position of nitrogen on the C-H bond where a radical is created when the hydrogen is removed reacting and forming hydroperoxides. These then have a cascading effect resulting in the formation of oxidation products [34]. 

**Figure 5:**
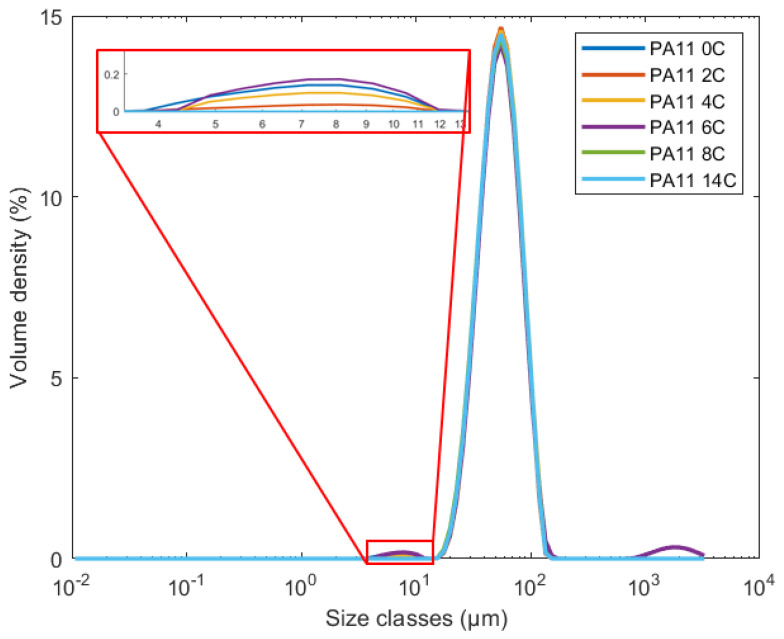
PSD curves for all stages of PA11 powder.

Figure 5 shows the representative curve for the powder size distribution (PSD) measurements for each stage of the powder, and Table 2 shows the characteristic particle sizes in the 10th, 50th, and 80th percentiles. The main curves appear to remain similar for the different aging stages of the PA11 powder. A difference can be observed at the lower sizes, around 10 μm, where all powders present a secondary peak with small volume of particles; however, that of PA11_14C and for PA11_6C presents a third peak at larger particle sizes above 10^3^ μm.

The characteristic particle sizes for the 10th and 50th percentiles only slightly vary among the different aging stages of the powder, while a decrease in the particle sizes of the 80th percentile after 24 h of exposure may be observed. This could be the result of fracturing caused by the evaporation of volatiles in the oven, as it is unlikely that the powder was completely free of moisture prior to aging. However, to conclusively understand the mechanism, moisture content measurements may be considered in the future. As previously found by Pandelidi et al. [9], variances in PSD can also effect the performance of the produced components as they affect the powder flowability and compaction during manufacturing.

**Table 2:** Characteristic powder particle sizes at the 10th, 50th, and 80th percentile for all stages of PA11 powder as measured though PSD.

	x_10.0_ (μm)	x_50.0_ (μm)	x_80.0_ (μm)
PA11_0C	31.2 ± 0.1	53.4 ± 0.2	87.6 ± 0.4
PA11_2C	31.7 ± 0.0	53.8 ± 0.1	75.1 ± 0.3
PA11_4C	31.7 ± 0.1	54.2 ± 0.2	75.8 ± 0.3
PA11_6C	31.4 ± 0.2	54.2 ± 0.9	77.0 ± 2.7
PA11_8C	31.2 ± 0.2	53.5 ± 0.1	75.2 ± 0.3
PA11_14C	31.5 ± 0.1	53.7 ± 0.1	75.3 ± 0.2

**Figure 6:**
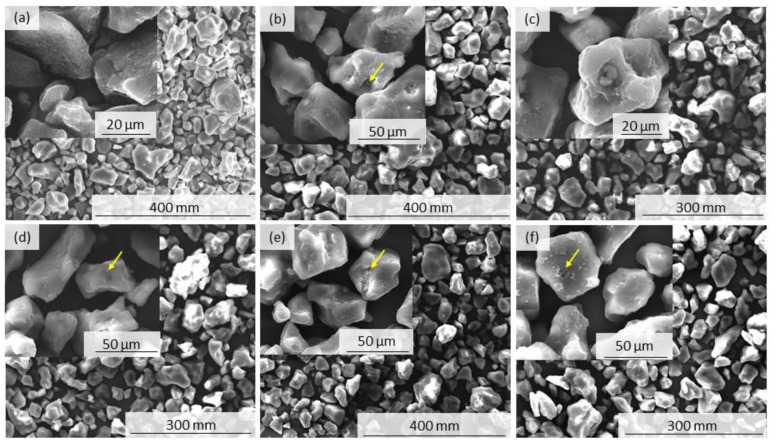
SEM images of (**a**) PA11_0C, (**b**) PA11_2C, (**c**) PA11_4C, (**d**) PA11_6C, (**e**) PA11_8C, and (**f**) PA11_14C powder particles. Yellow arrows showing powder particle cracking.

Figure 6 shows the morphology of the powder particles as captured using SEM. The PA11 powder particles appear to have the same convex shape as previously observed by Pandelidi et al. [9]. The particles of the PA11_0C powder have a smooth surface with no cracks or agglomerates. After 24 h of exposure, however, some powder particle cracking is observed, some examples of which are highlighted with yellow arrows. Similar cracking on the powder particles surface has previously been observed for powders used in PBF processes, but as other processing effects have taken place, their cause has been inconclusive [11]. In this research, though, the powder particles were not exposed to PBF processing which would have caused repetitive expansion and shrinkage phenomena; therefore, we suggest that the observed cracking is the result of the evaporation of volatiles. The agglomeration of particles was also observed after exposure, as seen in Figure 6c. This could explain the absence of a peak for small particles around 10 μm as observed in Figure 5 for the PA11_14C powder. 

These results demonstrate that both chemical and physical degradation take place on the PA11 powders with an increase in exposure time at 180 °C in air.

### 3.2. Thermal Analysis

**Figure 7:**
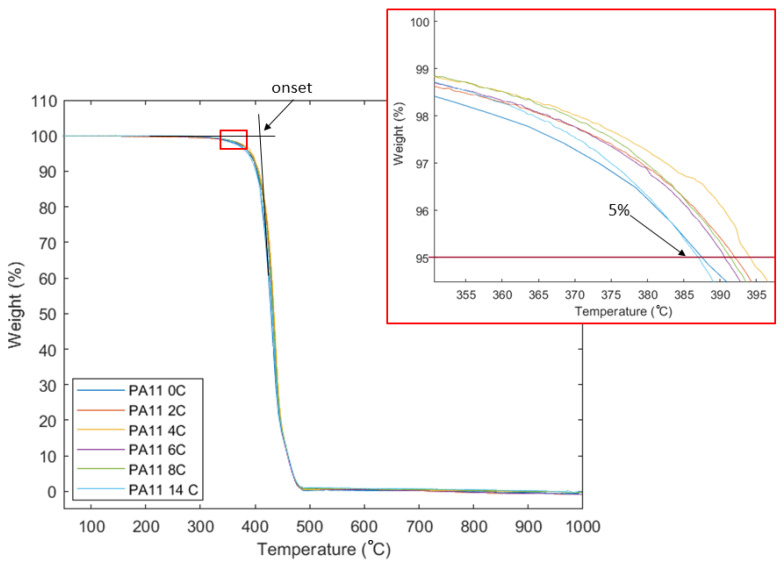
TGA curves and T_d_ at onset and 5% weight loss for all stages of PA11 powder.

TGA is used in this research to identify changes in T_d_ caused by phenomena such as post-polymerisation, chain scission, and crosslinking [15]. Figure 7 shows representative TGA curves for all stages of aged PA11. It needs to be noted early on, that it is believed that chain scission and post-polymerisation often occur at the same time [7,23] but due to the presence of oxygen during the aging process it is expected that chain scission will be the predominant aging mechanism in this research [7]. Previously, Okamba-Diogo et al. [21] found a low yield of chain scission at the early stages of thermal–oxidative aging of PA11 films which they explained was due to the presence of an efficiently competitive process through a series of complex chemical reactions that take place during aging. This means that thermal and rheological analysis may only present the mechanism prevailing at each stage, but do not exclusively suggest that the observed mechanism is the only one occurring. 

The average T_d_ at 5% weight loss and onset degradation of two specimens from each sample were measured by Proteus software. The curves show single stage decomposition, which is the result of decomposition due to scission of the main chain [35]. This is observed with all curves with some variance in the degradation onset temperatures. The onset T_d_ was found to slightly increase up to 48 h of exposure to 417 °C and decrease after that down to 413 °C after 168 h of exposure. PA11_0C was measured to have the lowest T_d_ at 5% weight loss at 385 °C, which increased gradually until the highest T_d_ for PA11_4C at 394 °C and then decreased again to 387 °C for PA11_14C. Both suggest that, against expectation, post-polymerisation might be the predominant mechanism for up to 48 h of exposure, after which point chain scission starts dominating the aging. However, this unexpected observation could be explained by the evaporation of volatiles that might have taken place up to 48 h delaying weight loss during TGA up to 48 h of exposure. This may be supported by the cracking observed on the surface of the powder particles shown in Figure 6b–f. 

Variances in glass transition temperature (T_g_) represent the brittle to ductile transition in the amorphous region of a semicrystalline polymer while T_m_ and T_c_ are used to examine their crystalline region. In this research, the T_g_ was measured for compression-moulded specimens through DMA, while DSC was used to measure the T_m_ and T_c_ of the powders. All thermal properties are listed in Table 3. 

**Figure 8:**
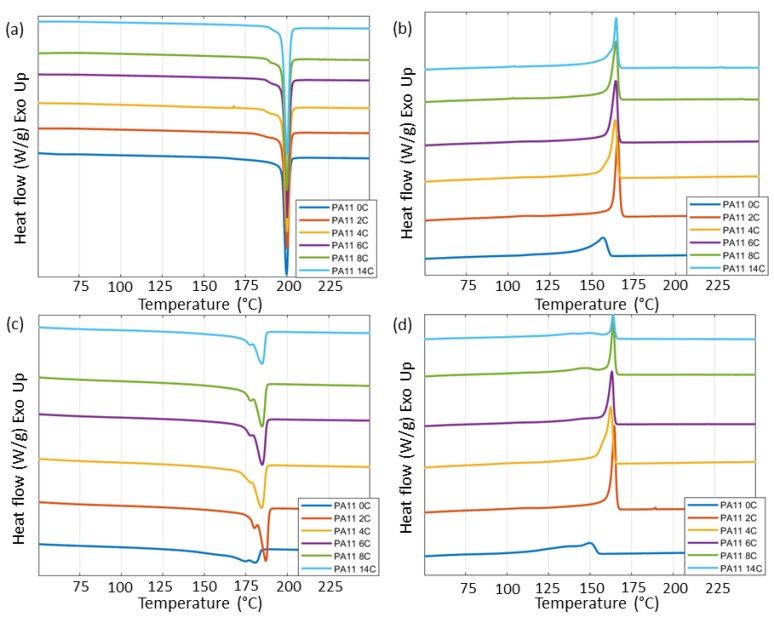
DSC curves for all stages of PA11 powder. (**a**) First-cycle melt peaks, (**b**) first-cycle crystallisation peaks, (**c**) second-cycle melt peaks, and (**d**) second-cycle crystallisation peaks.

Changes in T_m_ and T_c_ were expected indicators of powder deterioration due to aging [10]. Figure 8 shows the first and second heating cycle DSC curves for all PA11 powder stages. The first heating and cooling cycles in DSC reveal the thermal history of the material, while removing that history for more accurate reading of the material properties during the second cycle [9,15]. In this research, first-cycle measurements were primarily affected by the cooling of the powder after each aging stage. This cooling was not strictly controlled but it was kept as consistent as possible for each cycle. The measurements for PA11_0C were taken as baseline since this powder was as received from the powder supplier. In the first cycle, T_m_ was measured at 200 °C for all stages of the powder while small variances up to 2 °C were measured for the T_c_. Of greater interest are the observations in the thermal properties during the second cycle, where the thermal history among samples is equivalent, thus allowing for a direct comparison [15].

Considering the second-cycle heating results of Figure 8c, the morphology of the curves remains consistent with two melting peaks reflecting multiple spherulite cores with differed melt temperatures [15]. This suggests that no different lamellae are formed due to aging phenomena. This agrees with previous observations made by Okamba-Diogo et al. [5]. A 5 °C increase in the main T_m_ was measured between PA11_0C and PA11_2C, from which point on remained relatively constant while a fluctuation of 2 °C was measured for the secondary peaks at lower temperatures. Riedelbauch et al. [15] observed such an increase in T_m_ and expressed that it is the results of post-polymerisation or cross-linking phenomena taking place due to aging in PA12. These results agree with our observations and are consistent with those for T_d_. 

From the second cooling cycle curves of Figure 8d, a 6 °C increase in T_c_ was measured after 24 h of aging, which then gradually increased up to 96 h of exposure. A shift in the T_c_ to higher temperatures may be caused by chemicrystallisation, where fractions of the polymer chain resulting from chain scission may act as nucleating agents [36]. The post-polymerisation phenomena, indicated to have taken place based on the TGA results and the second heating cycle T_m_, would have resulted in a shift in the T_c_ to lower temperatures. This is because longer polymer chains resulting from post-polymerisation or cross-linking are expected to reduce the chain mobility, thus hindering crystallisation [15]. Riedelbauch et al. [15] in their study of PA12 powder also observed a similar trend of increasing T_m_ and T_c_ with increasing aging time, but they attributed these effects to subsequent treatment of the powder residue of the detailing agent from MJF processing [15]. Based on the TGA measurements of the detailing agent presented by Pandelidi et al. [9], this explanation is probable, however does not apply to the findings of this research as detailing agent was not introduced. 

At early stages of exposure of up to 48 h, an increase in crystallinity has previously been attributed to annealing, while increase in crystallinity at later stages above 100 h of exposure has been discussed to be the result of chemicrystallisation [5]. In Table 3, the crystallinity of the powders measured during the first heating cycle may reflect variances in the cooling rates after collection from the oven, while the crystallinity of the second heating cycle shows irreversible aging effects. The lower crystallinity measured by the second heating cycle when compared with that of the first heating cycle is probably a result of the relatively fast cooling during DSC and can also explain the lower T_m_ measured during the second heating cycle [9]. An increase in crystallinity, such as that observed here up to 96 h of thermo-oxidative aging, could be associated with polymer oxidation [5]. Such oxidation has been evident in the XPS results of this research. In semicrystalline polymers, such as PA11, oxidation is expected to primarily occur in the amorphous region, but even though the core of the crystallites is known to be inert to such effects, the boundary regions of those crystallites may also be affected [23,28].

**Table 3:** Thermal properties. DMA measured T_g_ for all stages of the compression-moulded specimens, T_m_, T_c_, and crystallinity as measured from DSC for all stages of PA11 powder, and T_d_ for 5% weight loss and at onset degradation as measured through TGA for all stages of PA11 powder.

	T_g_ (°C)	T_m_ (°C)		T_c_ (°C)		T_d_ (°C)		Crystallinity (%)	
		1st	2nd	1st	2nd	5%	Onset	1st	2nd
PA11_0C	63	200	179	164	154	385	411	63	26
PA11_2C	61	200	184	163	160	391	416	57	27
PA11_4C	57	200	184	163	161	394	417	60	31
PA11_6C	61	200	185	164	163	389	416	56	31
PA11_8C	60	200	185	165	164	390	413	61	32
PA11_14C	58	200	185	164	163	387	413	57	24

Small variances were found in the T_g_ of the specimens as well, with the T_g_ decreasing for specimens between PA11_0C and PA11_4C and then increasing for PA11_6C from which point it started decreasing again up to PA11_14C. PA11_0C noted the highest T_g_ at 63 °C and PA11_4C the lowest at 57 °C. A higher T_g_ suggests a greater chain entanglement, often the result of longer chains [36]. These observations would suggest that chain scission has taken place upon exposure, which is in-line with expectations.

The T_d_ of new PA11 powder has previously been reported at 394 °C and the melting peak of the first heating cycle at 198 °C, when heated at the same rate [9]. These minor inconsistencies in the reported values from the literature could be due to the different batches of the powder or due to different equipment being used during measurements. In this research, it was observed that the thermal properties of PA11 mainly varied between PA11_0C and PA11_2C, with minor changes beyond that point and up to 168 h of exposure, as reported by Riedelbauch et al. [15]. However, we would be inclined to suggest that changes as observed through two measurements of each sample group in TGA and DSC, are not sufficient to conclusively propose the aging mechanism of PA11 powder. Therefore, to investigate this further, we conducted rheology, DMA, and mechanical testing.

### 3.3. Rheological Properties

**Figure 9:**
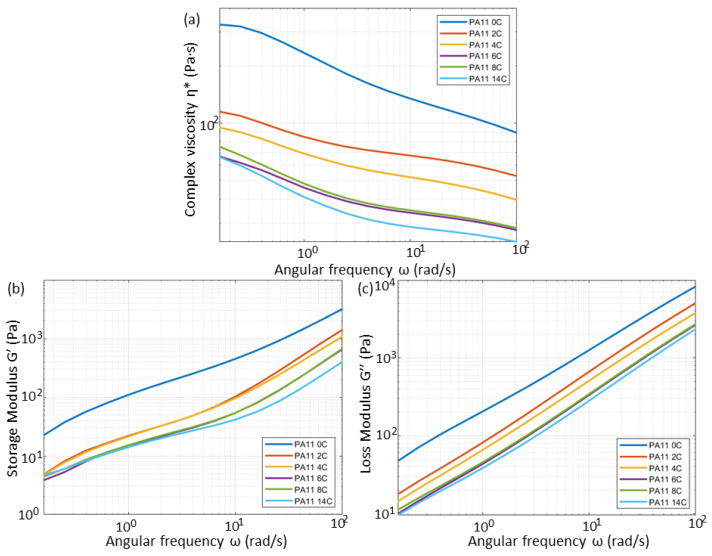
Parallel plate rheology curves for (**a**) complex viscosity, (**b**) storage modulus, and (**c**) loss modulus for all PA11 compression-moulded disks.

To better understand the aging mechanism among the aging stages of PA11, parallel plate rheology was performed, the results of which are shown in Figure 9. Valko et al. [6] expressed that the degradation mechanism in the presence of oxygen is expected to predominantly be that of scission, while in an inert atmosphere crosslinking is more probable. Figure 9a shows a decrease in η* with exposure time with a notable variance between PA11_0C and PA11_2C. This decrease in η* may be related to chain scission phenomena as it is typically an indicator of decreased chain entanglement [37]. This is reinforced by the fact that PA11_0C has greater shear thinning behaviour than its aged counterparts. This is observed by its more pronounced decrease in η* with increasing angular frequency. 

Similarly, G′ and G″ also decrease with exposure time with notable differences between PA11_0C and PA11_2C, while changes between PA11_2C and PA11_4C and among PA11_6C, PA11_8C and PA11_14C being minor.

### 3.4. Mechanical Properties

**Figure 10:**
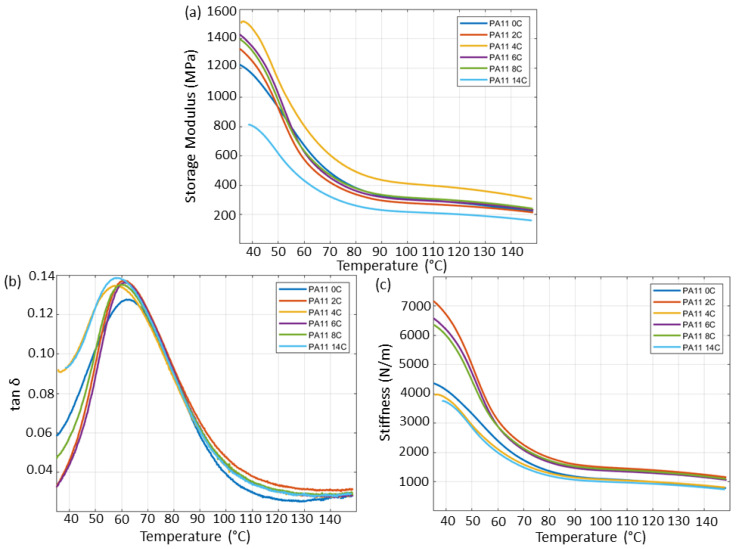
DMA curves for (**a**) storage modulus, (**b**) tanδ, and (**c**) stiffness for all compression-moulded PA11 bars.

Since the mechanical properties were measured using compression-moulded specimens, it is noted that further physical aging phenomena, such as molecular relaxation, are expected to have taken place [28]. Figure 10 shows the storage modulus, stiffness, and loss factor (tanδ) of PA11 specimens at various stages of thermal aging as measured by DMA. The storage modulus represents the elastic component of a viscoelastic polymer, such as PA11, and is associated with its stiffness, whereas tanδ shows the dumping properties of the polymer [38].

An increase in storage modulus was observed with thermal exposure, reaching a maximum after 96 h of aging. A decreasing trend in the storage modulus subsequently occurs with continued thermal exposure, with the storage modulus of PA11_14C reduced to a level below that of PA11_0C. All specimens show a monotonic decrease in storage modulus with increasing temperature associated with the softening of the material caused by the increased mobility of the polymer’s chains.

The intensity of the tanδ was lowest in the unaged specimen. While samples aged for 24 h and longer show a distinctly greater tanδ peak intensity than PA11_0C, the differences between the aged samples were unremarkable. The greater tanδ peaks are indicative of overall reduced elastic properties in the samples because of thermal aging. T_g_ measured from the tanδ peaks are presented in Table 3.

Below the T_g_, the storage modulus of PA11 increased after aging for 24 h and further after 48 h reaching its greatest value. After this point, a decrease in storage modulus may be observed from PA11_6C and PA11_8C, which remained above that of PA11_2C, and after 168 h of exposure, PA11_14C shows a notable decrease in storage modulus. The sharp decline in storage modulus observed around 60 °C is associated with the softening of the materials above it T_g_ caused by the increased mobility of the polymer chains. 

The stiffness of aged PA11 specimens, in Figure 10c, shows an increase after 24 h of exposure, a sharp decrease after 48 h and another increase after 72 h of exposure, from which point on an increase is observed. The stiffness of semicrystalline polymers is not directly dependent on their molecular weight, but a function of the degree of crystallinity. As crystallinity decreases, so does the stiffness and consequently the chances of brittle failure [39]. However, as previously discussed, crystallinity as a result of chemicrystallisation, rather than as a result of processing conditions such as cooling rates, may be related to molecular weight. 

**Figure 11:**
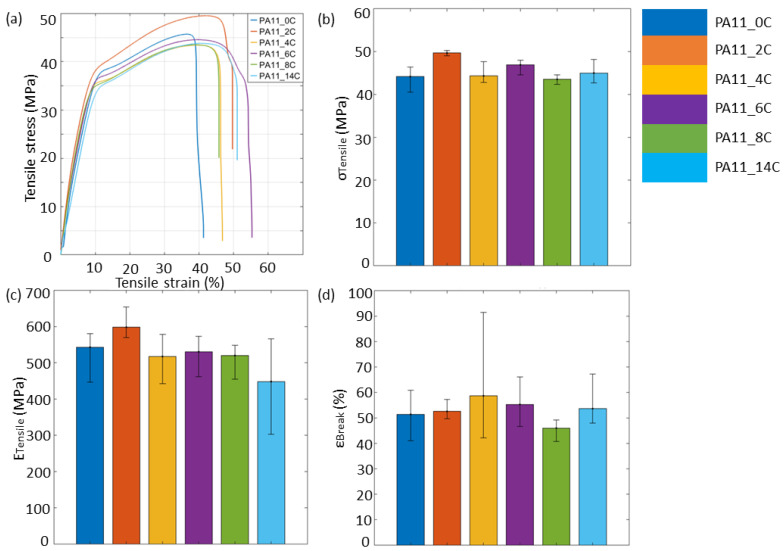
Tensile results for all compression-moulded Type I PA11 specimens. (**a**) stress–strain curves, and bar charts for (**b**) tensile strength, (**c**) Young’s Modulus, and (**d**) elongation at break with error bars.

As stated earlier, polymer aging may result in the deterioration of mechanical performance. Figure 11 shows representative stress–strain curves for all stages of PA11 specimens and the mean tensile strength (σ_Tensile_), E_Tensile_, and ε_Break_ with error bars. From Figure 11b, PA11_2C yielded the greatest σ_Tensile_ of 49.48 ± 0.60 MPa while PA11_8C had the lowest σ_Tensile_ of 43.53 ± 0.79 MPa. The σ_Tensile_ of polymers depends on different morphological characteristics, such as degree of crystallinity, molecular weight distribution and the type and size of crystals [40,41]. From the observations regarding crystal morphology based on the thermal analysis presented in this research and due to minimal variations in manufacturing of specimens, it is suspected that crystals remained the same in size and type through the aging stages. However, parallel plate rheological results indicated that the molecular weight distribution changed due to chain scission as early as after 24 h of thermo-oxidative aging;hence, it is suspected that this along with resulting changes in the degree of crystallinity may be responsible for the observed changes in σ_Tensile_. 

From the stress–strain curves of Figure 11a, all specimens failed in a ductile manner. It has been previously found that discolouration, such as that observed for the specimens in Figure 2, may be linked to embrittlement [5,23]. One of the mechanisms leading to embrittlement is the decrease in molecular weight due to chain scission usually in the amorphous region of semicrystalline polymers. Embrittlement as a result of chain scission is not only related to the minimised extension of the main chain due to reduction in length, but also to increase in crystallinity [39]. Chain scission has previously been found to be predominant mechanism over crosslinking in PA11 films [2] and is also suggested to be the case in the aged powder after 24 h of exposure. However, a continuous increase in embrittlement with exposure time was not observed in Figure 11d where PA11_8C has the lowest ε_Break_ at 46.00 ± 3.17%, whereas the greatest ε_Break_ was measured for PA11_4C specimens at 58.70 ± 19.85%. 

E_Tensile_ is a material property that depends on the elongation of the polymer chains along the loading direction [41]. In Figure 11c, small variances in E_Tensile_ among the sample groups can be observed. The greatest E_Tensile_ was that of PA11_2C at 598.4 ± 34.5 MPa and the lowest one measured for PA11_14C at 448.3 ± 104.4 MPa. The overall trends in E_Tensile_ with increasing thermal exposure are in good agreement with the trend observed in stiffness measured by DMA, as presented Figure 10c. A correlation between E_Tensile_ and T_g_ as presented in Figure 11c and Table 3, respectively, possibly suggests that E_Tensile_ is determined by the polymer chain configuration within the amorphous region. Our observations are in good agreement with Yao et al. [42], where the authors similarly reported an increase in E_Tensile_ and σ_Tensile_ of SLS PA2200 following exposure to high temperatures, before the properties decrease to close to virgin levels with continued thermal exposure. They attributed this phenomenon to the influence of thermal exposure on molecular chain complexity and weight increase, and presence of physical defects in the aged powder. Ding et al. [43] discussed that the E_Tensile_ of the amorphous phase is regulated by the Van der Waals forces that exist between polymer chains. As a result, significant reduction in the E_Tensile_ may only be observed when those forces are removed through chain scission. Through their calculations, though, they demonstrated that E_Tensile_ oscillates with the increasing number of chain scissions with a general descending trend [43], which is in line with the observations made in Figure 9a and Figure 11c. 

## 4. Conclusions

In this research, PA11 powder was exposed to thermo-oxidative aging for up to 168 h, and its physicochemical, morphological, thermal, rheological, and mechanical characteristics were evaluated as a function of exposure time. These analyses were performed to understand the effects of pre-fabrication aging as it is becoming increasingly relevant for PBF AM technologies on the performance of fabricated specimens without taking into consideration any process specific factors. 

It was found that the starting materials and the resulting compression-moulded specimens were affected by the aging process. The chemistry on the powder particle surface was found to be affected by the increased presence of oxygen and SEM revealed changes on the morphology of those particles. Through PSD, it was found that the size of larger particles decreased with aging, which may affect powder flowability and as a result part porosity during MJF. Even though the aged PA11 powders also underwent thermal analysis, the results were inconclusive as shifts in T_m_ were not in line with those in T_c_ and crystallinity. Instead, parallel plate rheology analysis was more suitable, which revealed that chain scission occurs as early as after 24 h of exposure. This is in line with existing reports in academic literature. DMA results agreed with observations from tensile testing. Tensile testing revealed that chemical characteristics, polymer chain configuration, and crystallinity may all affect the performance of specimens. However, a linear trend in mechanical properties with aging time was not observed, which may be attributed to the competing effects of these characteristics, as has previously been recognised. It is, therefore, understood that the exposure of PA11 powders in heat through the MJF process, where powders undergo thermo-oxidative aging as soon as after only two manufacturing cycles, will lead to the fabrication of components whose mechanical behaviour would be challenging to predict. These effects are not only related to the morphological effects of ageing on the size and shape of the powder particles impacting manufacturability, but also on its effect on the molecular structure and the impact on mechanical performance. From a practical perspective, increasingly pronounced yellowing of the material after just two cycles may limit powder reuse for aesthetic purposes. Therefore, manufacturing non-critical components with aged powder then applying a coating to mask the yellowing can be one approach to managing waste powder generation. 

## Figures and Tables

**Table 1:** Chemical composition of chemical bonds and C-N to C=O ratio from XPS and carbonyl ratio as measured from FTIR for all stages of the PA11 powder.

	C1s (at%)				O1s (at%)		C-N (at%)/C=O (at%)	Carbonyl Ratio (A1718/A1193)
	C-H	C-N	C=O	C-O	C=O	C-O		
PA11_0C	55.89	29.29	8.81	6.01	21.92	78.08	1.3362	0.03427
PA11_2C	63.99	21.52	8.58	5.91	24.60	75.40	0.8748	0.05150
PA11_4C	65.40	20.52	8.45	5.63	31.06	68.94	0.6606	0.04943
PA11_6C	66.16	19.30	8.65	5.90	32.74	67.26	0.5895	0.05229
PA11_8C	62.98	21.92	8.51	6.59	34.42	65.58	0.6368	0.06694
PA11_14C	59.46	24.79	8.56	7.18	40.67	59.33	0.6095	0.06746

## Data Availability

All data is contained within this article.
